# Effects of the SGLT2 inhibitor dapagliflozin on HDL cholesterol, particle size, and cholesterol efflux capacity in patients with type 2 diabetes: a randomized placebo-controlled trial

**DOI:** 10.1186/s12933-017-0529-3

**Published:** 2017-04-04

**Authors:** Gian Paolo Fadini, Benedetta Maria Bonora, Giancarlo Zatti, Nicola Vitturi, Elisabetta Iori, Maria Cristina Marescotti, Mattia Albiero, Angelo Avogaro

**Affiliations:** Department of Medicine, University of Padova, Via Giustiniani 2, 35128 Padua, Italy

**Keywords:** Atherosclerosis, Body composition, Therapy

## Abstract

**Background:**

Sodium-glucose co-transporter-2 inhibitors (SGLT2i) reduce glucose levels, body weight, and blood pressure, possibly resulting in cardiovascular protection. In phase III trials, SGLT2i were shown to increase HDL cholesterol. We aimed to evaluate whether the SGLT2i dapagliflozin affects HDL function in a randomized placebo-controlled trial.

**Methods:**

Thirty-three type 2 diabetic patients were randomized to receive dapagliflozin 10 mg or placebo for 12 weeks on top of their glucose lowering medications. The primary end-point was the change in cholesterol efflux capacity (CEC) from macrophages at study end versus baseline. Secondary endpoints were changes in: distribution of HDL subfractions, lipid profile, activity of enzymes that mediate HDL antioxidant properties (PON1 and ARE) and cholesterol metabolism (CETP), HbA1c, body weight and composition.

**Results:**

Thirty-one patients completed the study, n = 16 in the placebo group and n = 15 in the dapagliflozin group. Patients randomized to dapagliflozin were older and had lower adiposity indexes, although these differences disappeared after correction for multiple testing. Therapy with dapagliflozin reduced HbA1c by 0.9% and body weight by 3.1 kg, mainly attributable to reduction of body water and lean mass. As compared to placebo, dapagliflozin reduced CEC (−6.7 ± 2.4 versus 0.3 ± 1.8%; p = 0.043), but this effect was no longer significant after adjusting for age and BMI. No change was detected in HDL cholesterol, HDL subfractions, activity of PON1, ARE, and CETP.

**Conclusions:**

Despite improvements in glucose control and reduction in body weight, therapy with dapagliflozin exerted no significant effect on HDL cholesterol levels and HDL functionality.

*Trial registration* EudraCT 2014-004270-42; NCT02327039

**Electronic supplementary material:**

The online version of this article (doi:10.1186/s12933-017-0529-3) contains supplementary material, which is available to authorized users.

## Background

The pharmacologic armamentarium for the treatment of type 2 diabetes (T2D) has dramatically expanded in the last decade [1]. In parallel, cardiovascular protection has become one of the objectives of therapy [2], because cardiovascular diseases account for the majority of diabetes-related mortality [3]. Glucose-lowering medications differ for mechanism of action and side effects, and some drugs are also provided with ancillary cardiovascular benefits [1]. A great interest is devoted to the study of such extra-glycemic effects, and how they may translate into cardiovascular protection [2]. Furthermore, as some glucose-lowering medications have shown to increase the risk for major adverse cardiovascular events (MACE), regulatory agencies require that all new medications demonstrate safety in the pre- and/or post-marketing phase [4], including cardiovascular outcome trials [5].

Inhibitors of sodium-glucose co-transporter-2 (SGLT2i) prevent resorption of glucose from the proximal renal tubules, thereby inducing glycosuria and lowering glycemia. The amount of glucose lost with urine results in a significant reduction of body weight. As glucose exerts osmotic action, glycosuria is accompanied by an increased urinary output and a reduction in blood pressure [6]. In phase III randomized clinical trials (RCTs) the SGLT2i dapagliflozin was found to reduce HbA1c by about 0.6–0.9%, body weight by about 2–3 kg, and blood pressure by about 3–5 mmHg [7].

In a ground-breaking cardiovascular outcome trial, the SGLT2i empagliflozin was superior to placebo in reducing the rate of MACE, mortality, and hospitalization for heart failure [8]. During the trial, differences in HbA1c, body weight and blood pressure in patients on empagliflozin versus those on placebo were too small to explain the magnitude of cardiovascular protection [9]. This suggested that other, mostly unknown, mechanisms may be responsible for the observed benefit. Several speculative theories have been proposed but none has been validated experimentally [10–12].

In phase III RCTs, as well as in the EMPA-REG Outcome trial [8], therapy with SGLT-2i has been associated with a decrease in serum triglycerides, an increase in HDL cholesterol, and a small increase in LDL cholesterol [13–16], although this has not always been confirmed by real world data [17]. Diabetic dyslipidemia, which is characterized by raised triglycerides and low HDL cholesterol levels, is one contributor to the high cardiovascular risk of T2D [18]. Several medications commonly used in T2D patients may affect the lipid profile [19] and a therapeutic increase in HDL cholesterol may explain, at least in part, cardiovascular protection by SGLT-2i.

Despite HDL cholesterol is inversely associated with cardiovascular risk, levels of cholesterol contained in HDL particles is an imprecise measure of the anti-atherosclerotic effects of HDL, which is mainly mediated by reverse cholesterol transport and anti-oxidant activity [20]. Indeed, cholesterol efflux capacity (CEC) from macrophages, a metric of HDL-mediated reverse cholesterol transport, is more strongly associated with atherosclerosis than HDL cholesterol levels [21].

In this study, we specifically aimed to evaluate the effects of dapagliflozin on CEC, HDL sub-fractions, and activity of HDL-associated anti-oxidant enzymes.

## Methods

### Study design and objectives

This was a randomized, placebo-controlled, phase IV clinical trial. The protocol was approved by the local ethical committee (Prot. 3302/Ao/14) and by the National competent authority, and registered on http://www.clinicaltrials.gov (NCT02327039). All procedures involving human subjects were carried out in accordance with the Declaration of Helsinki.

The objective of the study was to evaluate the effects of dapagliflozin on HDL particle size and function. The primary end-point was the change versus baseline in CEC by patients’ serum after therapy with dapagliflozin compared to placebo. Secondary end-points were the changes versus baseline in the following parameters: HDL cholesterol levels; distribution of HDL subclasses; HDL anti-oxidant activity; CETP activity; circulating concentrations of hormones and inflammatory mediators.

Patients were recruited between April 2015 and June 2016 from the diabetes outpatient clinic of the University Hospital of Padova. All consecutive patients with the required demographic characteristics were screened. Inclusion criteria were: females or males aged 18–75 years, diagnosis of T2D with a disease duration of at least 6 months, underlying therapy with oral glucose-lowering medications and/or insulin. Patients had to be on a stable statin dose for at least 3 months prior to study entry or be at LDL cholesterol target. Exclusion criteria were: acute illness or infection; recent (within 1 month) surgery, trauma, or cardiovascular event; recent (within 3 months) variation of statin therapy/dose; alcoholism; very high baseline HDL cholesterol levels (>90 mg/dl); previous history of recurrent (≥2 episodes) urinary tract infections or genital infections (a single remote episode not to be considered an exclusion criterion); history of hypotension or frequent episodes of volume depletion/dehydration; chronic kidney disease (eGFR <60 ml/min/1.73 mq); chronic liver disease (SGOT or GPT >twofold ULN, or cirrhosis); heart failure, NYHA classes III-IV; hypersensitivity to dapagliflozin or its excipients; ongoing treatment with pioglitazone or GLP-1 receptor agonists; pregnancy or lactation, inability to provide informed consent. All patients provided written informed consent prior to any study specific procedure.

For all patients, we recorded the following baseline characteristics: age, sex, body mass index, waist circumference, systolic and diastolic blood pressure, smoking habit (defined as habitual active smoking of 1 or more cigarettes per day), lipid profile (total, HDL and LDL cholesterol, and triglycerides), serum creatinine, urinary albumin/creatinine ratio (mg/g). The estimated glomerular filtration rate (eGFR) was calculated with the CKD-EPI formula [22] and graded according to the KDOQI (Kidney Disease Outcomes Quality Initiative) [23]. Diabetic retinopathy was defined based on digital fundus photography scored remotely by expert ophthalmologists and graded according to the Early Treatment of Diabetic Retinopathy Study (ETDRS) [24]. Somatic peripheral neuropathy was defined, after exclusion of non-diabetic causes, in the presence of typical sensory or motor symptoms (numbness, tingling, or pain in the toes, feet, legs, hands, arms, and fingers, or wasting of the muscles of the feet or hands), confirmed by clinical examination (ankle reflexes, vibratory perception threshold, pinprick, and 10-g monofilament sensitivity) and eventual determination of neural conduction velocity. Coronary artery disease (CAD) was defined as a history of myocardial infarction or angina, or evidence of significant coronary artery disease at coronary angiography. Peripheral arterial disease (PAD) was defined as a history of claudication or rest pain, or significant stenosis in leg arteries. Asymptomatic atherosclerosis was defined as the presence of carotid artery plaques (stenosis >15%) at routine ultrasound examination.

### Randomization, blinding and treatment

After obtaining informed consent, patients were randomized to receive dapagliflozin 10 mg (the standard clinically approved dose in Italy) or placebo, based on a computer-generated unpredictable sequence. For safety reasons, the study was single blind meaning that patients, but not the clinical study staff, were unaware of the allocated treatment. However, the study staff in charge of the primary and secondary end-point evaluation was kept blind, thereby avoiding any interference on the study results. To guarantee concealment, pills and dispensers of dapagliflozin and placebo were identical.

At the beginning of the study, patients accessed the outpatient clinic at 8:00 am and fasting blood samples were collected. Aliquots of plasma and serum were stored at −80 °C until analysis. Systolic and diastolic blood pressure were measured in the sitting position, after at least 15 min rest, using the same calibrated manometer throughout the study. Body weight was recorded while the patient was wearing light dressing using the same instrument throughout the study. The analysis of body composition was performed using the BIA system (Akern BIA 101) and the BIA software. Bioelectrical impedance vector analysis (BIVA) was performed as described by Piccoli and Pastori (BIVA software. Department of Medical and Surgical Sciences, University of Padova, Padova, Italy, 2002, available from apiccoli@unipd.it).

Patients were instructed to take dapagliflozin or placebo pills daily in the morning on top of their previous glucose-lowering regimen. Patients taking insulin or sulphonylureas were allowed to down-titrate such medications to avoid hypoglycaemia. Treatment duration was 12 weeks. Patients then returned to the clinic for blood sampling, determination of body weight and composition, systolic and diastolic blood pressure. Compliance to study medications was evaluated by counting pills remained in the returned dispenser. Information on eventual side effects were also recorded.

### Analytical measures

#### Cholesterol efflux capacity

Cholesterol efflux capacity (CEC) was quantified using a slightly modified method designed to increase throughput [25]. The protocol was optimized to increase the yield of CEC and to be related to HDL functionality. Details are given in the Additional file 1: Appendix.

#### Lipid profile and lipoprotein particles

Serum total cholesterol, HDL cholesterol, LDL cholesterol (direct method) and triglycerides concentrations were measured using a Roche automated modular analyser COBAS 8000. In addition, LDL cholesterol levels were also estimated using the Friedwald’s formula. The distribution of HDL lipoproteins into subfractions based on particle size was analysed using the Lipoprint system, according to the manufacturer’s instructions. Briefly, the system uses high resolution polyacrylamide gel electrophoresis that separates and measures the amount of cholesterol in each LDL and HDL subfraction.

#### HDL antioxidant activity

PON1 activity was measured with a commercially available kit (EnzChek^®^ Paraoxonase Assay Kit, Molecular Probes, Inc. USA) according to the manufacture’s instruction. 10 µl of serum was incubated with a fluorogenic organophosphate analog and fluorescence (excitation/emission 360/450 nm) was read continuously at 37 °C for 60 min. We calculate PON1 activity by interpolating kinetic data with the standard curve provided with the assay. Levels of arylesterase (ARE) activity were determined according to the protocol described in Huen et al. [26]. Rate of formation of phenol was monitored in a Beckman^®^ DU530 spectrophotometer every 15 s (270 nm, ambient temperature) after the addition of 40 μl (1:40 dilution) of serum to 400 μl of a 3.26 mmol/l phenyl acetate solution (9 mmol/l Tris–HCL pH 8.0 0.9 mmol/l CaCl2).

#### CETP activity

CETP assay was performed with a commercially available kit (BVN-K595-100, BioVision, California, USA), which measured the fluorescence generated after the transfer of self-quenched fluorescent neutral to an acceptor molecule. Fluorescence is proportional to the amount of neutral lipid transferred. Samples were diluted and after 30 min of incubation with the substrate, fluorescence (Ex/Em = 480/511 nm) was read continuously for xx minutes to generate a kinetic curve. The standard curve provided with the kit allows to calculate CETP activity.

#### Hormones and inflammatory mediators

Plasma concentrations of adiponectin, GIP, GLP-1 glucagon, visfatin, resistin, leptin, TNF-α, IL-6, IL-8, and PAI-1 were quantified using a custom multiplex suspension array that allows the simultaneous immuno-detection of several analytes in a single well. Biomarkers were sampled with Bio-Plex^®^ Multiplex Immunoassay kits (Bio-Rad Laboratories, USA) according to the manufacture’s instruction. For Cytokines and Diabetes assays, serum was diluted 1:4 while for the Adiponectin assays, serum was diluted 1:400 and the test run separately. All the reagents and the standard curves were prepared according the manufacture’s instructions. The assays were performed on Luminex^®^ 200™ instrument.

### Statistical analysis

Continuous data are expressed as mean ± standard error if normal, or as median (interquartile range) if non-normal. Normality was checked using the Shapiro–Wilk test. Non-normal data were log transformed before analysis. Categorical data are presented as percentage. Comparison between two groups were performed using unpaired Student’s *t* test or the Fisher’s exact Chi square test where appropriate. Variables collected at study end were compared to data at baseline using the paired Student’s t test. Study end-points were evaluated by calculating within-group changes versus baseline, which were then compared between the two groups. Correction for confounders was performed using multiple linear regression models wherein changes in outcome variables were entered as dependent variables. SPSS software (IBM) version 24.0 was used. Statistical significance was accepted at p < 0.05.

Sample size was determined for the primary endpoint variable. Based to our previous experience in a similar setting and a similar population of T2D patients [27, 28], we calculated that n = 15 patients/group were sufficient to detect a significant 15% difference versus baseline in cholesterol efflux capacity (absolute value 1.2 AU) with sigma = 1.1 AU, alpha = 0.05, beta = 0.20.

## Results

### Patient characteristics

**Fig. 1:**
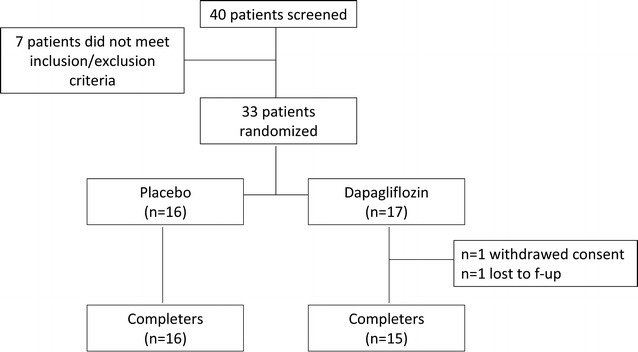
Study flow-chart with number of patients screened, randomized and completers

A total of 33 patients were enrolled, who were randomly assigned to dapagliflozin (n = 17) or placebo (n = 16). Two patients in the dapagliflozin group dropped out: one withdrew before initiating investigational drug and one was lost to follow-up. Thus, n = 31 patients completed the study, n = 15 allocated to dapagliflozin and n = 16 to placebo. As none of the completers withdrew investigational drug, an intention to treat analysis was performed for all completers, which corresponds to the per protocol analysis (Fig. 1). Compliance to investigational drug, as determined by residual pill counting was high and similar between placebo (91.4 ± 1.6%) and dapagliflozin (92.3 ± 1.6%; p = 0.705). Clinical characteristics of completers are shown in the Table 1. Despite randomization, patients assigned to dapagliflozin therapy were older and leaner. Owing to the large number of variables collected, these differences may be the result of chance and indeed were no longer significant after adjusting for multiple testing.

**Table 1:** Clinical characteristics of study subjects

Variable	All (n = 31)	Placebo (n = 16)	Dapagliflozin (n = 15)	*p*
Demographics and anthropometrics				
Age, years	63.7 ± 1.3	61.0 ± 1.8	66.3 ± 1.8	0.034^a^
Sex male, %	67.7	66.7	68.8	1.000
BMI, kg/m^2^	30.5 ± 1.0	32.8 ± 1.4	28.4 ± 1.4	0.018^a^
Waist, cm	107.7 ± 2.2	111.7 ± 3.0	103.3 ± 3.0	0.049^a^
HbA1c, %	8.2 ± 0.1	8.2 ± 0.2	8.2 ± 0.2	0.908
Diabetes duration	14.1 ± 1.2	13.9 ± 1.3	14.2 ± 1.3	0.916
Concomitant risk factors				
Hypertension, %	90.3	93.3	87.5	1.000
Smoke, %	19.4	20.0	18.8	1.000
Total cholesterol, mg/dl	163.3 ± 6.6	158.1 ± 11.1	168.1 ± 11.1	0.458
HDL cholesterol, mg/dl	48.5 ± 2.2	47.2 ± 3.3	49.7 ± 3.3	0.587
LDL cholesterol, mg/dl	89.2 ± 6.0	82.3 ± 10.0	95.6 ± 10.0	0.275
Triglyceridex, mg/dl	137.1 ± 16.2	141.0 ± 19.5	133.4 ± 19.5	0.818
ACR, mg/g	68.2 ± 45.9	116.4 ± 94.1	22.9 ± 94.1	0.316
Creatinine, mg/dl	0.82 ± 0.03	0.81 ± 0.05	0.83 ± 0.05	0.794
eGFR (ml/min/1.73 mq)	90.8 ± 3.0	92.5 ± 4.4	89.3 ± 4.4	0.603
Complications				
Retinopathy, %	25.8	33.3	18.8	0.433
Nephropathy, %	22.6	33.3	12.5	0.220
Neuropathy, %	3.2	6.7	0.0	0.484
CAD, %	25.8	40.0	12.5	0.113
PAD, %	0.0	0.0	0.0	1.000
CerVD, %	58.1	53.3	62.5	0.722
Medications				
Metformin, %	93.5	100.0	87.5	0.484
SU, %	19.4	20.0	18.8	1.000
Glinides, %	3.2	0.0	6.3	1.000
DPP-4i, %	19.4	13.3	25.0	0.654
Basal insulin, %	16.1	13.3	18.8	1.000
Basal-bolus insulin, %	35.5	46.7	25.0	0.273
ACEi/ARB, %	80.6	86.7	75.0	0.654
Other anti-hypertensive, %	61.3	73.3	50.0	0.273
Statins, %	90.3	100.0	81.3	0.226
Anti-platelet, %	51.6	60.0	43.8	0.480

^a^Not significant after correction of type I error

### Effects on HbA1c, blood pressure, body weight and composition

HbA1c levels were well balanced at baseline between the 2 groups. After 12 weeks, HbA1c was 8.6 ± 0.4% in the placebo group and 7.3 ± 0.4% in the dapagliflozin group (p = 0.004). At study end, HbA1c was <7.0% in 1/15 patients (6.7%) who had received placebo and 6/16 patients (37.5%) who had received dapagliflozin (p = 0.03). Change in HbA1c versus baseline was 0.4 ± 0.2% in the placebo group and −0.9 ± 0.2% in the dapagliflozin group (p < 0.0001). The age and BMI adjusted placebo-corrected change in HbA1c was −1.1 ± 0.3%. Change in body weight was 0.1 ± 0.5 kg in the placebo group and −3.1 ± 0.5 kg in the dapagliflozin group (p = 0.0001). The age and BMI adjusted placebo-corrected change in body weight was −2.9 ± 0.8 kg. Significantly more patients in the dapagliflozin than in the placebo group achieved the composite endpoint of HbA1c <7.0% and no weight gain (37.5 versus 0.0%; p = 0.001).

Systolic blood pressure declined by 4.7 ± 1.3 mmHg in the dapagliflozin group and by 1.0 ± 2.3 mmHg in the placebo group (p = 0.035). Diastolic blood pressure declined by 1.3 ± 0.6 mmHg in the dapagliflozin group and by 0.4 ± 1.6 mmHg in the placebo group (p = 0.317).

The analysis of body composition by bio-impedenzometry showed that dapagliflozin, as compared to placebo, significantly reduced fat-free mass (−2.9 ± 1.3 versus 0.1 ± 1.3 kg; p = 0.047) and total body water (−2.4 ± 1.0 l versus 0.04 ± 1.0 l; p = 0.041), but had no effect on fat mass (−0.1 ± 1.4 versus −0.4 ± 1.4 kg; p = 0.806). Changes in fat-free mass (p = 0.190) and total body water (p = 0.172) were no longer significant after adjustment for age and BMI. BIVA analysis showed a displacement of the mean vector towards dehydration in dapagliflozin-treated patients, whereas no change was observed in patients who received placebo (Additional file 1: Figure S1).

### Safety

During the study, 1 patient in the placebo group reported dizziness, and 3 patients in the dapagliflozin group developed symptoms suggestive of genito-urinary tract infection. These adverse events did not lead to discontinuation of investigational drug and resolved spontaneously or after study conclusion and drug withdrawal. The hematocrit increased by 1.5 ± 0.8% in the dapagliflozin group and by 1.2 ± 0.6% in the placebo group (p = 0.708).

### Primary endpoint: cholesterol efflux capacity

Cholesterol efflux capacity was evaluated as the ability of patients’ serum to extract fluorescently labeled cholesterol from cultured macrophages, using a customized protocol optimized to yield information on HDL reverse cholesterol transport. At baseline, there was no significant difference in cholesterol efflux capacity between the two groups. At study end, cholesterol efflux was reduced by 6.7 ± 2.4% in the dapagliflozin group and by −0.3 ± 1.8% in the placebo group (p = 0.043). The age and BMI adjusted placebo-controlled change in CEC was −5.2 ± 3.3% (p = 0.126).

### Secondary endpoints

#### Lipid profile

We detected no changes in total cholesterol, HDL cholesterol, LDL cholesterol and triglycerides in both the dapagliflozin and placebo groups (Table 2).

**Table 2:** Lipid profile, HDL subfractions, cholesterol efflux capacity and enzymatic activity at baseline and study end in the two groups

Variable	Placebo (n = 16)			Dapagliflozin (n = 15)		
	Baseline	12 weeks	Change	Baseline	12 weeks	Change
Lipid profile						
Total cholesterol	150.6 ± 9.8	151.7 ± 6.2	1.1 ± 6.4	162.2 ± 5.8	169.9 ± 11.2	7.7 ± 9.2
HDL cholesterol	46.3 ± 3.0	47.4 ± 3.5	1.0 ± 1.1	48.1 ± 3.4	46.8 ± 3.8	−1.4 ± 2.3
LDL cholesterol (direct method)	75.8 ± 8.1	76.5 ± 5.4	0.9 ± 5.4	86.3 ± 4.1	92.5 ± 9.5	6.2 ± 8.2
LDL cholesterol (Friedewald formula)	76.4 ± 8.1	76.5 ± 5.4	0.1 ± 6.1	85.9 ± 5.2	95.0 ± 9.7	9.1 ± 9.5
Triglycerides	139.1 ± 13.9	138.9 ± 14.5	−0.1 ± 12.2	141.1 ± 19.8	140.9 ± 18.5	−0.1 ± 17.9
HDL subfractions, %						
Large	27.8 ± 2.2	27.8 ± 2.1	0.0 ± 1.7	24.8 ± 2.8	23.9 ± 2.6	−0.9 ± 1.3
Intermediate	51.5 ± 1.2	51.3 ± 0.9	−0.3 ± 1.1	54.2 ± 1.9	53.5 ± 1.6	−0.7 ± 1.1
Small	20.3 ± 2.8	21.0 ± 2.3	0.7 ± 1.7	20.7 ± 2.1	22.6 ± 1.9	1.9 ± 1.0
HDL subfractions, mg/dl						
Large	13.6 ± 1.5	13.5 ± 1.7	−0.1 ± 1.0	11.7 ± 1.7	11.7 ± 1.6	0.0 ± 0.6
Intermediate	24.9 ± 1.9	23.9 ± 1.9	−1.1 ± 1.2	25.2 ± 2.1	25.3 ± 2.1	0.0 ± 0.7
Small	9.5 ± 1.2	9.5 ± 1.1	0.0 ± 0.8	9.4 ± 0.9	10.5 ± 1.0	1.2 ± 0.6
Enzymatic activity						
PON1, U/ml	46.1 ± 6.4	50.3 ± 7.6	4.2 ± 4.9	40.8 ± 3.9	41.5 ± 2.9	0.7 ± 2.7
ARE, kU/ml	138.8 ± 22.1	138.9 ± 17.0	0.1 ± 24.8	156.3 ± 21.1	149.4 ± 17.2	−7.0 ± 30.9
CETP, mU/ml	1.4 ± 0.1	1.3 ± 0.1	0.0 ± 0.1	1.1 ± 0.1	1.1 ± 0.1	0.0 ± 0.1
Cholesterol efflux capacity, %	35.6 ± 2.7	35.3 ± 2.2	−0.3 ± 1.8	40.8 ± 2.8	34.2 ± 2.2*^#^	−6.7 ± 2.4*

* *p* < 0.05 versus placebo

^#^ *p* < 0.05 versus baseline

#### HDL particle size

The Lipoprint system was used to determine the distribution of HDL particles within 10 subfractions based on size. No significant changes were detected in the placebo group and in the dapagliflozin group in the distribution in 10 subfractions, or in 3 fractions (small, intermediate and large) when analyzed as percentage distribution, or cholesterol content (Table 2).

#### HDL anti-oxidant capacity and CETP activity

We found no change in PON1 and ARE activity from baseline to 12 weeks in both groups, and no changes in CETP activity (Table 2).

#### Circulating biomarkers

Plasma biomarkers, including cytokines, adipokines and hormones were measured using a multiplex based array. Patients who received dapagliflozin showed a significant reduction in visfatin concentrations compared to baseline, significantly lower (Table 3) end-of-treatment levels of IL-6 compared to patients receiving placebo, and a significant placebo-subtracted reduction in leptin concentrations. All these effects lost statistical significance after adjusting for age and BMI. In the whole study cohort, changes in leptin and in IL-6 concentrations were significantly correlated with absolute or percent change in body weight.

**Table 3:** Levels of cytokines, hormones and inflammatory mediators in the two groups at baseline and after 12 weeks

	Placebo (n = 16)			Dapagliflozin (n = 15)		
	Baseline	12 weeks	Change	Baseline	12 weeks	Change
Adiponectin (μg/ml)	19.2 ± 3.7	20.1 ± 3.7	0.8 ± 5.0	22.6 ± 5.3	18.7 ± 3.6	−3.9 ± 4.0
GIP (pg/ml)	349.7 ± 71.7	353.9 ± 75.0	4.2 ± 41.0	393.5 ± 58.3	366.7 ± 78.0	−26.8 ± 68.0
GLP-1 (pg/ml)	194.2 ± 43.3	216.5 ± 41.7	22.4 ± 20.4	167.5 ± 25.3	156.3 ± 23.6	−11.3 ± 22.4
Glucagon (pg/ml)	323.5 ± 61.2	277.6 ± 55.2	−45.9 ± 49.4	270.3 ± 57.7	281.8 ± 33.6	11.5 ± 38.2
Visfatina (ng/ml)	2.8 ± 0.6	3.1 ± 0.3	0.3 ± 0.4	1.8 ± 0.4	2.8 ± 0.6^#^	1.0 ± 0.4
Resistina (ng/ml)	13.7 ± 0.9	16.0 ± 1.7	2.3 ± 1.9	13.4 ± 1.5	14.3 ± 2.1	0.9 ± 1.8
Leptin (ng/ml)	9.6 ± 1.7	10.5 ± 1.9	0.9 ± 0.6	7.8 ± 1.8	6.9 ± 1.5	−0.9 ± 0.6*
IL-6 (pg/ml)	3.2 ± 0.8	4.0 ± 0.8	0.8 ± 1.1	3.3 ± 0.8	1.8 ± 0.5*	−1.6 ± 0.8
IL8 (pg/ml)	8.3 ± 1.2	13.7 ± 3.7	5.4 ± 4.0	9.3 ± 2.0	8.2 ± 0.9	−1.1 ± 1.9
TNF-α (pg/ml)	3.3 ± 1.1	5.4 ± 1.4	2.0 ± 1.6	5.0 ± 1.2	5.4 ± 1.4	0.2 ± 0.9
PAI-1 (ng/ml)	21.3 ± 1.2	23.4 ± 1.2	2.0 ± 1.3	20.3 ± 1.1	20.6 ± 1.4	0.4 ± 1.2

* *p* < 0.05 versus placebo

^#^ *p* < 0.05 versus baseline

## Discussion

This trial was designed to test the effects of dapagliflozin on HDL levels, distribution, and function. The rationale was based on the observation that, in phase III clinical trials, SGLT2i increased HDL cholesterol levels [13]. Formally, the study succeeded in detecting a significant change in the primary end-point, CEC, which was reduced by dapagliflozin as compared to placebo. This is predicted to translate into a pro-atherosclerotic effect, as cholesterol efflux from macrophage is part of the reverse cholesterol transport that antagonize cholesterol accumulation within the artery wall [21, 29]. However, no change occurred in HDL cholesterol levels, HDL particle size, and activity of enzymes that modulate HDL antioxidant properties (PON1 and ARE) and cholesterol metabolism (CETP). Although the determinants of CEC are partially unknown, inflammation, lipid composition, and HDL particle size have been related to CEC in several populations [30]. As dapagliflozin marginally reduced IL-6 and had no effect on lipid profile and HDL subfractions, the observed reduction in CEC has no clear explanation. A comparison between a priori estimated and a posteriori calculated statistical power suggests that this finding, although significant, may be poorly reproducible. The study had an estimated 80% power to detect a significant 15% difference versus baseline in CEC, whereas the observed difference was equal to 16.6% of baseline and calculated power was 47%. Furthermore, baseline CEC tended to be higher in patients randomized to dapagliflozin as compared to those randomized to placebo, although this difference was not statistically significant. In fact, despite randomization, there were some imbalances in baseline patient characteristics, such as age and adiposity indexes. CEC decreased significantly only in the dapagliflozin group and change from baseline was significantly larger than in the placebo group, but end-of study values were similar in the two groups. As baseline values and changes over time in CEC were highly inversely correlated (r = −0.65; p < 0.001), our finding may even represent a regression to the mean, rather than an effect of dapagliflozin. In fact, when CEC change in dapagliflozin versus placebo-treated patients was adjusted for age and BMI, which differed at baseline in the two groups, statistical significance disappeared. To account for some between-group imbalance in baseline clinical characteristics, we also ran a multiple regression analysis wherein all confounding variables shown in Table 1 with a *p* value <0.5 were entered as covariates together with the assigned treatment: no effect of dapagliflozin versus placebo was noted for CEC, HDL cholesterol or HDL subfractions (not shown).

In addition to these statistical considerations, other study results have to be taken into account to interpret the findings on lipid levels and HDL function. As compared to placebo, dapagliflozin therapy reduced HbA1c by 1.3% and body weight by 3.2 kg. The effect on HbA1c was larger than in most RCTs [31] because patients randomized to placebo experienced a worsening in glycemic control. Intuitively, a significant decline in body weight is expected to be accompanied by improvements in the lipid profile, as observed with GLP-1 receptor agonists [32–34]. It is also noteworthy that the effects on HDL may be differ according to the ethnic group, as observed for metformin [35].

The analysis of body composition by BIA showed that weight loss was associated with loss of lean mass and total body water, but not fat mass. Similar results have been obtained with 8-week tofogliflozin treatment in Japanese T2D patients using BIA [36]. In addition to the estimation of fat and lean mass, the vector analysis can be applied to bioelectric impedance data [37]. This analysis confirms that the main effect of dapagliflozin was a reduction in body fluid content. This finding contrasts with the reduction in leptin concentrations observed in the dapagliflozin versus the placebo group, which would imply a reduction in fat mass [38]. In addition, studies using dual-energy X-ray analysis (DEXA) have shown reduction of fat mass after 24–104 weeks of dapagliflozin therapy [39, 40]. If BIA data are reliable, we speculate that dapagliflozin therapy may take longer to cause a reduction in fat mass, which may then translate into improvements in the lipid profile. It is indeed noteworthy that even triglyceride levels were unaffected by dapagliflozin in this study, despite a significant reduction in body weight and an improvement in glucose control. However, BIA mainly measures total body water, whence lean mass is estimated, and then fat mass is calculated by difference from total body weight. It has been shown that BIA overestimates fat mass in case of reduction in extracellular volume [41]. Thus, the diuretic effect of SGLT-2 inhibition with dapagliflozin may have led to an overestimation of fat mass at the end of the study. This is one possible reason why BIA-estimated fat mass was not reduced at study end in the dapagliflozin group. In summary, we speculate that our study was unable to detect a change in fat mass after dapagliflozin therapy either because a treatment duration >12 weeks is needed to achieve such effect, or because of the technical limitations of BIA versus DEXA.

## Conclusions

In summary, we show that, despite a remarkable improvement in glucose control and reduction in body weight, a 12-week therapy with dapagliflozin exerted no significant effects on HDL cholesterol levels and HDL functionality. These data do not support that the beneficial cardiovascular effects of SGLT-2i is mediated by modifications of HDL.

## Additional file

**Additional file 1.** Supplemental data on cholesterol efflux capacity and bioimpedance vector analysis.

## Abbreviations

ACEi: angiotensin converting enzyme inhibitors
ACR: albumin creatinine ratio
ARB: angiotensin receptor blocker
ARE: arylesterase
AU: arbitrary unit
BIA: bioelectrical impedance analysis
BIVA: bioelectrical impedance vector analysis
BMI: body mass index
CAD: coronary artery disease
CEC: cholesterol efflux capacity
CerVD: cerebrovascular disease
CETP: cholesteryl ester transfer protein
CKD: chronic kidney disease
DEXA: dual-energy X-ray analysis
DPP-4i: dipeptydil peptidase 4 inhibitor
eGFR: estimated glomerular filtration rate
ETDRS: Early Treatment of Diabetic Retinopathy Study
GIP: glucose dependent insulinotropic peptide
GLP-1: glucagon like peptide 1
HDL: high density cholesterol
IL: interleukin
KDOQI: Kidney Disease Outcomes Quality Initiative
LDL: low density cholesterol
MACE: major adverse cardiovascular events
NYHA: New York Heart Association
PAD: peripheral arterial disease
PAI: plasminogen activator inhibitor
PON1: paraoxonase 1
RCT: randomized controlled trial
SGLT2i: sodium glucose co-transporter inhibitor
SU: sulphonylurea
T2D: type 2 diabetes
TNF: tumor necrosis factor
ULN: upper limit of normal
