# Supplementary material for: Effects of the SGLT2 inhibitor dapagliflozin on HDL cholesterol, particle size, and cholesterol efflux capacity in patients with type 2 diabetes: a randomized placebo-controlled trial
Source: Cardiovasc Diabetol. 2017 Apr 4;16:42. doi: 10.1186/s12933-017-0529-3 (PMC5379610; doi:10.1186/s12933-017-0529-3)
Supplement: Supplementary file 1 — Additional file 1. Supplemental data on cholesterol efflux capacity and bioimpedance vector analysis. [file 12933_2017_529_MOESM1_ESM.doc]

**Supplementary Appendix**

**Cholesterol efflux capacity**

Cholesterol efflux capacity was quantified using a slightly modified method designed to increase throughput . We first verified that CEC of complete serum was 25% higher than that of plasma, and similar to that of purified HDL. J774 cells were seeded onto a 24 well plate at a density of 3x10^5^ cells and incubated at 37°C and 5% CO_2_ in RPMI 1640 medium containing 10% FBS, 2 mM glutamine, 1% Penicillin/Streptomycin (P/S). The following day, cells were washed with PBS and incubated for 1.30 h in 1% FBS RPMI 1640 medium containing 0.025 mM BODIPY-cholesterol (Avanti Polar Lipids Inc, Spectra 2000, Rome, Italy), 0.2% bovine serum albumin (BSA), 2 mM glutamine, 1% P/S, and 1 mM methyl-β-cyclodextrin (MβCD). Then, in order to quantify fluorescence cholesterol uptaken by the cells, the medium was transferred into clean tubes. J774 cells were equilibrated for 18h with serum-free RPMI 1640 medium, containing 0.2% BSA, 1% P/S, and 2 mM glutamine. ATP binding cassette transporter A1 (ABCA1) was up-regulated adding 0.3 mM 8-(4-chlorophenylthio)-cyclic AMP, to increase CEC. After this equilibration period, the cells were washed with PBS and incubated for 4h with fresh medium containing 10% sample serum. At the end of each step, 100 µl of medium were transferred onto 96 well black plates. The fluorescence intensity was measured using a Mithras LB 940 plate reader (Berthold Italia S.r.l. Brugherio-MB) (excitation 485 nm, emission 530 nm). All steps were performed in the presence of 2 μg /ml 8-(4-chlorophenylthio)-2’-O-methyladenosine (CI976), an acyl–coenzyme A:cholesterol acyltransferase (ACAT) inhibitor. Percent efflux was calculated by the following formula: fluorescence cholesterol intensity in medium containing 10% sample serum ÷ fluorescence-cholesterol intensity in cells before the efflux step × 100. Bodipy-cholesterol efflux to serum-free media (“background efflux”) was 4.9±1.8% and was subtracted from Bodipy-cholesterol effluxes of all the samples. In order to correct the inter-assay variation across plates, a pooled serum control was included on each plate. Each sample was run in duplicate, and values were normalized by dividing the efflux capacity of individual patients by the efflux capacity of a serum pool run with each assay. We verified a close correspondence between CEC determined by ^3^H-cholesterol and Bodipy-cholesterol loading (r=0.99). Cellular cholesterol loading in positive and negative controls was verified by dual-labelling experiments . After Bodipy cholesterol incubation for 4 h, cells were washed twice with PBS and exposed to 10 µl Hoechst 33342 (5 mg/mL) for 15 min to label DNA. For confirmation, fluorescent cholesterol and nuclei were visualized from living cells. Images were acquired with a 20x objective on a fluorescence microscope Axiovert 200M (Carl Zeiss Spa, Milan, Italy). All reagents and J774 macrophages were purchased from Sigma Aldrich, Milan, Italy, unless otherwise specified.

**References**

1. Sankaranarayanan S, Kellner-Weibel G, de la Llera-Moya M, Phillips MC, Asztalos BF, Bittman R, Rothblat GH: **A sensitive assay for ABCA1-mediated cholesterol efflux using BODIPY-cholesterol**. *J Lipid Res* 2011, **52**(12):2332-2340.

2. Liu Z, Thacker SG, Fernandez-Castillejo S, Neufeld EB, Remaley AT, Bittman R: **Synthesis of cholesterol analogues bearing BODIPY fluorophores by Suzuki or Liebeskind-Srogl cross-coupling and evaluation of their potential for visualization of cholesterol pools**. *Chembiochem* 2014, **15**(14):2087-2096.

3. Piccoli A: **Bioelectric impedance measurement for fluid status assessment**. *Contrib Nephrol* 2010, **164**:143-152.

**Supplemental Figure 1. Bioimpedance vector analysis (BIVA)**. A) The steady state graph (adapted from ) shows how to interpret the vector tolerance with respect to hydration status and fat/lean mass. Concentric ellipses represent 50%, 75% and 95% confidence intervals. The change in mean vector before and after treatment with placebo and dapagliflozin is represented in (B) and (C) respectively. The concentric ellipses indicate the entire study population. D) The vector displacement graph (adapted from ) shows how to interpret changes of the mean vector over time in the same subjects. The mean vector with 95% confidence ellipse for dapagliflozin and placebo are shown in (E). Panel F shows the mean displacement vector in patients who received dapagliflozin or placebo.
